# MRI Based Localisation and Quantification of Abscesses following Experimental *S*. *aureus* Intravenous Challenge: Application to Vaccine Evaluation

**DOI:** 10.1371/journal.pone.0154705

**Published:** 2016-05-26

**Authors:** Elizabeth R. Allen, Pauline van Diemen, Yuko Yamaguchi, Claudia Lindemann, Elizabeth Soilleux, Christine Rollier, Fergal Hill, Jurgen Schneider, David H. Wyllie

**Affiliations:** 1 Jenner Institute, Centre for Cellular and Molecular Physiology, University of Oxford, Oxford, United Kingdom; 2 Nuffield Department of Clinical Laboratory Sciences, Radcliffe Department of Medicine, University of Oxford, Oxford, United Kingdom; 3 Oxford Vaccine Group, Department of Paediatrics, University of Oxford, Oxford, United Kingdom; 4 The NIHR Oxford Biomedical Research Centre, Centre for Clinical Vaccinology and Tropical Medicine, University of Oxford, Oxford, United Kingdom; 5 Imaxio SA, Lyon, France; 6 BHF Experimental MR Unit, Radcliffe Department of Medicine, University of Oxford Oxford, United Kingdom; ContraFect Corporation, UNITED STATES

## Abstract

**Purpose:**

To develop and validate a sensitive and specific method of abscess enumeration and quantification in a preclinical model of *Staphylococcus aureus* infection.

**Methods:**

*S*. *aureus* infected murine kidneys were fixed in paraformaldehyde, impregnated with gadolinium, and embedded in agar blocks, which were subjected to 3D magnetic resonance microscopy on a 9.4T MRI scanner. Image analysis techniques were developed, which could identify and quantify abscesses. The result of this imaging was compared with histological examination. The impact of a *S*. *aureus* Sortase A vaccination regime was assessed using the technique.

**Results:**

Up to 32 murine kidneys could be imaged in a single MRI run, yielding images with voxels of about 25 μm^3^. *S*. *aureus* abscesses could be readily identified in blinded analyses of the kidneys after 3 days of infection, with low inter-observer variability. Comparison with histological sections shows a striking correlation between the two techniques: all presumptive abscesses identified by MRI were confirmed histologically, and histology identified no abscesses not evident on MRI. In view of this, simulations were performed assuming that both MRI reconstruction, and histology examining all sections of the tissue, were fully sensitive and specific at abscess detection. This simulation showed that MRI provided more sensitive and precise estimates of abscess numbers and volume than histology, unless at least 5 histological sections are taken through the long axis of the kidney. We used the MRI technique described to investigate the impact of a *S*. *aureus* Sortase A vaccine.

**Conclusion:**

Post mortem MRI scanning of large batches of fixed organs has application in the preclinical assessment of *S*. *aureus* vaccines.

## Introduction

*Staphylococcus aureus* is a commensal of the human skin and nares and a frequent cause of infection in humans[1]. Virulent, often multi-resistant clones are prevalent worldwide[2]. There is currently no licensed vaccine, and *S*. *aureus* vaccine development is ongoing in an attempt to reduce both antibiotic use driven by the organism, and its high economic and human burden[3]. Abscess formation in internal organs is a hallmark of severe invasive disease[4]. Established abscesses frequently require drainage, prolonged antibiotic therapy, or a combination of these[5]. Therefore, preventing the initiation or early progression of abscesses is a key goal of anti- *S*. *aureus* vaccination.

Abscess formation is a process which appears to be mediated by single organisms seeding tissues to which they gain access within phagocytes [6, 7]. There follows a multi-step process which includes rapid recruitment of neutrophils and macrophages to the site of bacterial proliferation[8]. Thus, the formation of abscesses can be viewed in terms both of abscess number and abscess size. Preclinical vaccine studies with *S*. *aureus* have used a mouse model involving intravenous *S*. *aureus* administration, where bacterial count from kidneys is a measure for efficacy [9]. Although recovery of viable bacteria from infected organs provides some information about vaccine efficacy, this may not necessarily reflect abscess number or progression[10]. In recognition of this, some studies have used in addition histological assessment of abscess formation[11], a laborious technique which only examines a limited part of the kidney, depending on the number of sections taken. These studies showed that bacterial counts increased little after day 2 or 3 of intravenous infection, although abscess size continued to increase[10].

Several studies in mice have investigated the effects of specific *S*. *aureus* genes on abscess formation in *in vivo* models [10, 12]. In these studies, *SrtA* deficienct bacteria were unable to establish abscesses, with elimination of *SrtA* deficient bacteria by day 15 post infection. Sortase A, encoded by *SrtA*, is a membrane bound transpeptidase which is a member of the group of microbial surface components recognizing adhesive matrix molecules (MSCRAMMs). Sortase A recognizes LPXTG and related motifs which are present on *S*. *aureus* proteins directed for membrane anchoring and surface display[13]. Of these LPXTG proteins, many have been reported to have a direct role in virulence and abscess formation, such as clumping factors A and B (ClfA, ClfB), staphylococcal protein A (SpA), serine-aspartate repeat protein D (SrdD) and fibronectin binding proteins (FnBpA and FnBpB)[10, 14]. Therefore, effective inhibition of sortase A by vaccines might therefore be expected to attenuate infection severity and abscess formation.

In recent years many studies have investigated the use of various imaging techniques such as MRI, biolumiscient imaging or PET scanning to detect bacterial infections[15–18].

Here we describe a technique to assess the formation of staphylococcal abscess(es) in murine renal tissue, which relies on post-mortem MRI of fixed organs embedded in agar. We could analyse 32 kidneys in a single run. We validate the technique against histological appearances, and illustrate its use in the assessment of efficacy of a *S*. *aureus* SrtA vaccine.

## Methods

### Bacteria and growth conditions

*Staphylococcus aureus* strain Newman wild type (wt) was obtained from Professor T. Foster, Trinity College, Dublin. Bacteria were routinely grown on Horse Blood Agar (HBA) plates (Oxoid, UK) or in tryptic soy broth (TSB, Oxoid, UK). Long-term storage of bacteria was at -80°C. Strain identity was checked by colony morphology, Gram stain, mannitol fermentation, coagulase production, DNAse activity, and sequencing of PCR products obtained across the *SpA* locus (data not shown). For infection of mice, 3–4 colonies of *S*. *aureus* were picked from HBA plates and transferred to TSB and grown overnight at 37°C, 130 rpm. This overnight culture was subcultured 1:100 into fresh TSB and grown statically for 2.5 h at 37°C. The culture was washed and resuspended in Phosphate Buffered Saline (PBS, Sigma Aldrich, UK) at approximately 1×10^8^ CFU/ml. The actual concentrations of all washed *S*. *aureus* cultures were verified by plating and colony enumeration.

### Animals

All mouse procedures were conducted in accordance to the Animal (Scientific Procedures) Act 1986 (Project licence 30/2825) and were approved by the University of Oxford Animal Care and Ethical Review Committee. For the experiments in this study, specific pathogen free female BALB/c mice, aged 6 weeks were obtained from Harlan Laboratories (Bicester, UK) and housed in individually filtered cages on a normal diet.

### Establishment of MRI technique in the Intravenous challenge model

Female BALB/c mice were housed in groups of 4. Twelve mice were inoculated i.v. in the lateral tail vein with 0.1 mL (~10^7^ CFU) bacterial suspension, prepared as above. Four mice were injected with 0.1 mL PBS as controls. Mice were observed daily throughout the course of the experiment. Individual mice that reached pre-defined humane endpoints as described in S1 Table before the set end of the experiment were culled immediately. On Day 3, 7 and 10 postinfection, mice were sacrificed (dislocation of the neck), and kidneys harvested. Viable *S*. *aureus* per gram tissue was enumerated by spirally plating dilutions of the homogenized left kidney on Horse Blood Agar (HBA) plates (Oxoid, UK) using Autoplate^®^ Automated Spiral Plater (Advanced Instruments, Inc., Norwood, MA, USA). Plates were read using QCount Automated Colony Counter (Advanced Instruments, Inc) or manually after 24 hour incubation at 37°C. The sensitivity of detection was taken to be 100 CFU/g. The right kidney was stored in 4% paraformaldehyde (PFA, Alfa Aesar, UK) for MR processing and imaging.

### Adenoviral vaccines used

DNA encoding SrtA, corresponding to amino acids 57–206 of WP_053875978.1 including an inactivating C184A mutation [19], was human codon optimised and synthetised by Life Technologies Ltd. using GeneArt^®^ Gene Synthesis (www.lifetechnologies.com/UK). This antigen construct was subcloned into a mammalian expression vector by restriction digestion and checked by sequencing. This plasmid expresses the protein of interest behind a mammalian leader sequence, fused at the C-terminus to a multimerising IMX313 tag[20]. pMono2 is a mammalian expression vector constructed in Oxford University for this purpose by modification of the vector pENTR4 (Invitrogen); construction details have been published in patent WO2014053861A2. From pMono2 the antigen construct was transferred into pAdHu5-DEST (Life Technologies) using Gateway technology (Life Technologies) and the resulting construct linearised by PacI digestion and transfected into replication-deficient adenovirus human serotype 5 (AdHu5) as described elsewhere [21, 22]. The control adenovirus was generated by transfer of the expression backbone (pMono2) without any transgene into pAdHu5-DEST.

### Production of SrtA protein

Recombinant SrtA-IMX313 (protein IMX501), corresponding to amino acids 60–206 of WP_053875978.1, was expressed from *E*. *coli* cells and purified by ion exchange chromatography. The resulting product was multimeric, and was >95% pure, as judged by 15% SDS-PAGE electrophoresis. Endotoxin concentrations were estimated at 250 EU/mg (Lonza LAL QCL-1000 assay).

### Assessment of efficacy of a *S*. *aureus* SrtA vaccine

Female BALB/c mice were housed in groups of 8. At 7 weeks old, mice received an intramuscular injection with 10^9^ i.u. human adenovirus 5 (AdHu5) expressing either SrtA, or no antigen, followed 8 weeks later by 20 μg SrtA protein (protein preparation reference IMX501) per mouse in AbISCO-100 (Isconova, Sweden, now acquired by Novavax, USA) or AbISCO-100 alone. At intervals, venous blood samples were taken from all animals for immune assays. Two weeks after the last immunisation mice were inoculated in the tail vein with 0.1 ml (~10^7^ CFU) bacterial suspension, as described above. The mice were observed daily throughout the course of the experiment, with humane end-points as described above. Fifteen days post infection mice were sacrificed. The kidneys were harvested and processed as described above.

### Assessment of Vaccine Immunity: Luciferase ImmunoPrecipitation System (LIPS)

A Luciferase ImmunoPrecipitation System (LIPS) assay was used to detect specific serum anti-*S*. *aureus* Sortase A antibodies as described [23]. Briefly, serially diluted serum was incubated with *Renilla* luciferase-Sortase A fusion protein. The mix was added to filter plates loaded with A/G beads (Thermo Fisher). After incubation and subsequent washings, chemoluminescence was measured in a Luminometer (VarioSkan Flash, Thermo Fisher) after adding substrate (Renilla luciferase assay system, Promega UK Ltd.). Log transformation was applied to luminescence data prior to statistical analysis. Specific luminescence was generated by subtracting the assay background, which was considered to be the luminescence observed in the absence of any sera. The assay limit of detection was considered to be four standard deviations above the specific luminescence in the control groups.

### Assessment of Vaccine Immunity: IFN-γ ELISpot

Individual peripheral blood samples were treated with ACK lysis buffer to remove red cells prior to stimulation with a pool of 20-mer peptides overlapping by 10, spanning amino acids 57–206 of SrtA at a final concentration of 2 μg/ml in the presence of homologous splenocytes (5x10^6^ cells/ml), on High Protein Binding Immobilon-P membrane plates (MAIPS4510, Millipore) coated with 5 mg/ml anti-mouse IFN- γ (AN18, Mabtech). After 18–20 hours incubation, IFN- γ spot forming cells (SFC) were visualised by staining membranes with anti-mouse IFN-γ biotin (1 μg/ml, R4-6A2, Mabtech) followed by streptavidin-Alkaline Phosphatase (1 μg/ml, Mabtech) and development with AP conjugate substrate kit (BioRad, UK). Number of SFC were counted with an ELISpot reader (AID, Germany). Log SFC was used in statistical analysis because of the approximate log-normal distribution of ELISpot counts in the animals [20].

### MRI scanning

Kidneys were removed from infected mice and fixed in 4% PFA (Alfa Aesar, UK). We found kidneys could be stored for long periods of time (up to 12 months tested) before imaging with no detrimental effect. Kidneys were removed from PFA and soaked in a gadolinium-based contrast agent (Omniscan, GE Healthcare) at a concentration of 0.5 mmol/ml for 24 hours, then mounted in a 28 mm glass tube and embedded in 1% agarose (Iberose, Web scientific, UK) containing 2mM gadolinium contrast agent.

The imaging sequence we routinely use in our lab for MR microscopy of fixed tissues and mouse embryos is based on a gradient echo pulse sequence, which generates a mixture of T1- (due to a high flip-angle of 60° and a tissue-dependent Gd-uptake) and T2*-contrast (long echo time of 10ms) [24]. In addition, the Gd helps to bring down the T1 times, allowing for shorter repetition times, and thus for shorter scan times. In particular, allowing for the data to be acquired in unattended overnight runs greatly maximises the usage of an (expensive) preclinical MR-system. While it was not specifically optimized for the detection of abscess in kidneys, the first test experiments yielded excellent resolution and contrast, demonstrating the applicability of our MRI technique to successfully differentiate between healthy kidney tissues and abscess.

Imaging experiments were carried out on a 9.4 T (400 MHz) MR system (Varian Inc., Palo Alto, USA) comprising a horizontal magnet (bore size 210 mm), a VNMRS Direct DriveTM console and an actively shielded gradient system (1000 mT/m, rise time 130 msec, od 115 mm, id 60 mm). A quadrature-driven birdcage RF-coil (Rapid Biomedical, Rimpar, Germany) was used for transmitting / receiving the MR signals. The MRI sequence was carried out essentially as previously described [25], using a fast spoiled 3D gradient echo sequence with T1-weighting (TE/TR = 10/ 30ms; flip-angle: 60°, matrix size 1408x608x608. field-of-view: 50x26x26mm, sagittal orientation, total scan time: 12.25h).

### Image analysis

Images were analysed using Amira software version 5.6 (FEI Visualisation Sciences Group). This software reconstructs 2D images from the 3D MRI in any plane, allowing the comparison with histological analysis conducted on the same tissue. Abscesses were identified visually by operators reviewing the MRI images simultaneously in three views (xy, xz, yz) by scrolling through ‘movie’ style, and then reviewing suspect areas. Criteria used to identify suspect areas include (i) loss of normal renal architecture around the lesion (ii) an area of altered density identified by histological comparison to reflect an abscess (iii) areas of segmental abnormality, suggestive of vascular infarction. These latter were noted, but were not considered to be part of abscess formation. A supplementary protocol provides additional details. Abscess volume was estimated using the label field tool in Amira software which allows the selection of abscesses from the MR image. The material statistics tool was applied to the labelled data which then calculates the volume of all labelled areas. Abscess identification and size estimation were measured by one or two operators independently, blinded to the treatments associated with the kidney studied.

### Histological analysis

Subsequent to MR scan, kidneys were removed from agar and stored in PFA. Following embedding in wax, three 5μM sections were taken through the long (sagittal) axis of each kidney at approximately 15%, 35% and 50% renal thickness and stained with haematoxylin and eosin staining in the Oxford Centre for Histological Research (OCHRE), John Radcliffe Hospital, Oxford.

### Statistical analysis

Data on renal bacterial load was analysed for effect of vaccination by means of a t-test after a log transformation of count data. To assess correlation between abscess areas, estimated by histology, and abscess volumes estimated by MRI, Spearman’s r was computed. GraphPad Prism v 5.04 (GraphPad Software, Inc.) was used for all computations.

## Results

Mice were inoculated with 10^7^ CFU *S*. *aureus* and were culled at days 3, 7 and 10 to harvest kidneys. One mouse scheduled for culling at day 10 had reached the pre-defined humane end-point of the experiment on day 7 and was therefore culled and used in analysis for the day 7 time point.

### MR imaging technique is able to detect and quantify abscess in murine kidney

**Fig 1:**
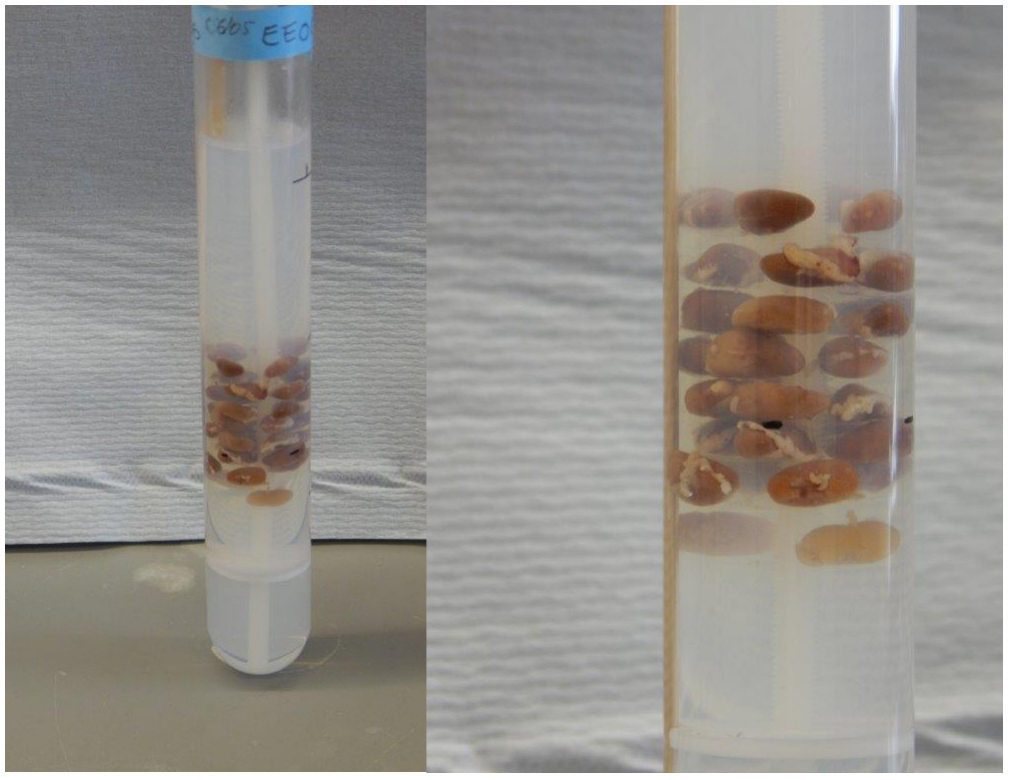
Scanning fixed organs after agar embedding. Up to thirty-two paraformaldehyde-fixed kidneys can be placed in eight layers of four kidneys, and embedded in agarose containing the contrast agent. The tube is then subjected to MR imaging.

We were able to image up to 32 kidneys in a single MRI run in an agar tube (Fig 1), yielding images at high spatial resolution (25.4 x 25.4 x 24.4μm) which afforded excellent definition of renal cortex, medulla, and collection systems (S1 Video, which shows a typical set of normal kidneys). Comparison of images obtained three days after infection (S2 Video) revealed obvious focal lesions, localised in the renal cortex. At later time points, these focal lesions increased in size (S3 Video, showing lesions at 7 and 10 days).

We developed a standardised technique for identifying and quantitating these lesions, which appeared compatible with *S*. *aureus* abscesses (S1 File). The technique relied on examination of MRI series, rather than single sections. Amira image analysis software was used to assist with detection and marking of lesion margins, and to compute their sizes. Two observers with no previous background in imaging were trained.

**Fig 2:**
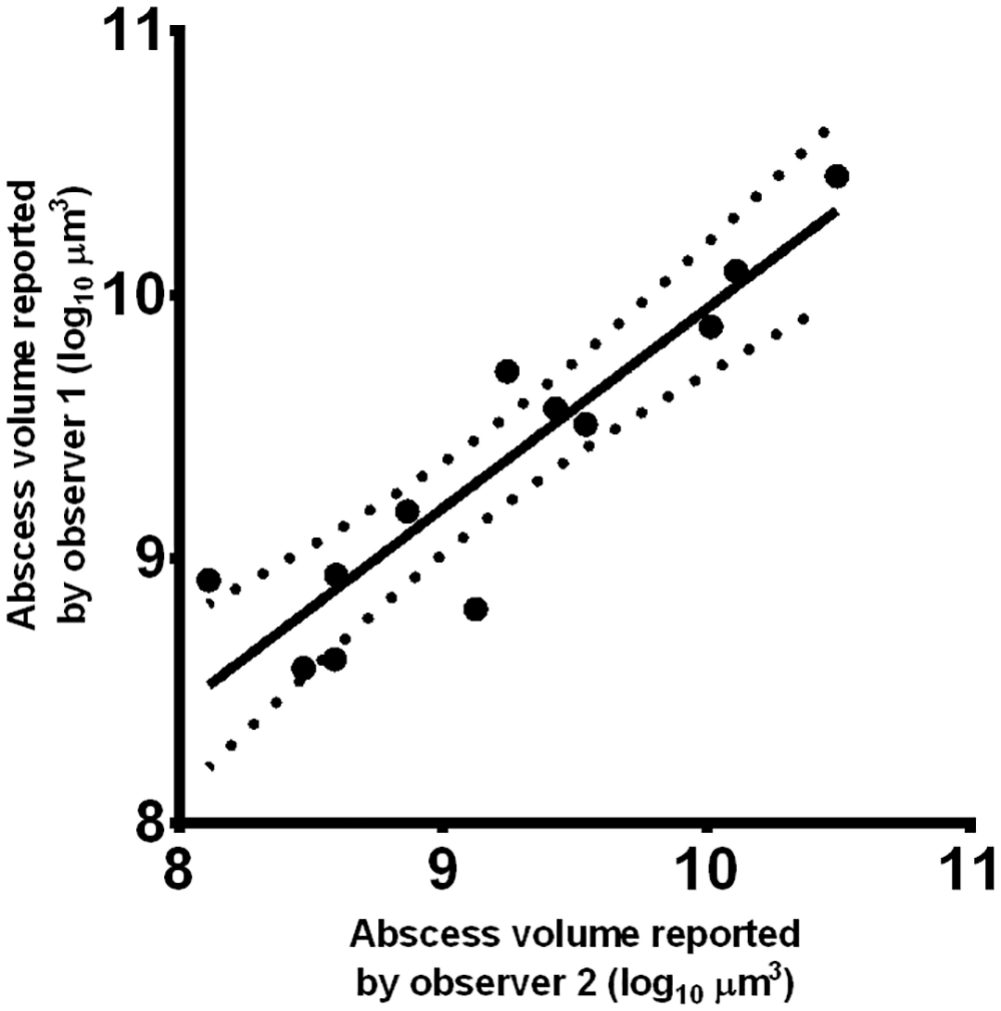
Inter-observer variability. Two observers were trained to use the MRI technique. After training, they were tested independently on a 16 kidney test data set. The total volume of abscesses in each kidney estimated by both observers is shown.

Subsequently, a 16 kidney test data set (S1–S3 Videos), consisting of 4 kidneys from each of four time points (uninfected, 3, 7, 10 days post infection), was assembled and scored independently, blind to group membership, by the two independent observers. Importantly, when the infected kidneys were analysed and compared blindly by two observers, there was a strong positive correlation in the abscess volumes as calculated by each observer (Pearson’s correlation coefficient = 0.915, p<0.005), Fig 2. We concluded the process of reading MRI scans was reproducible.

#### Validation of MRI technique by subsequent histology

**Fig 3:**
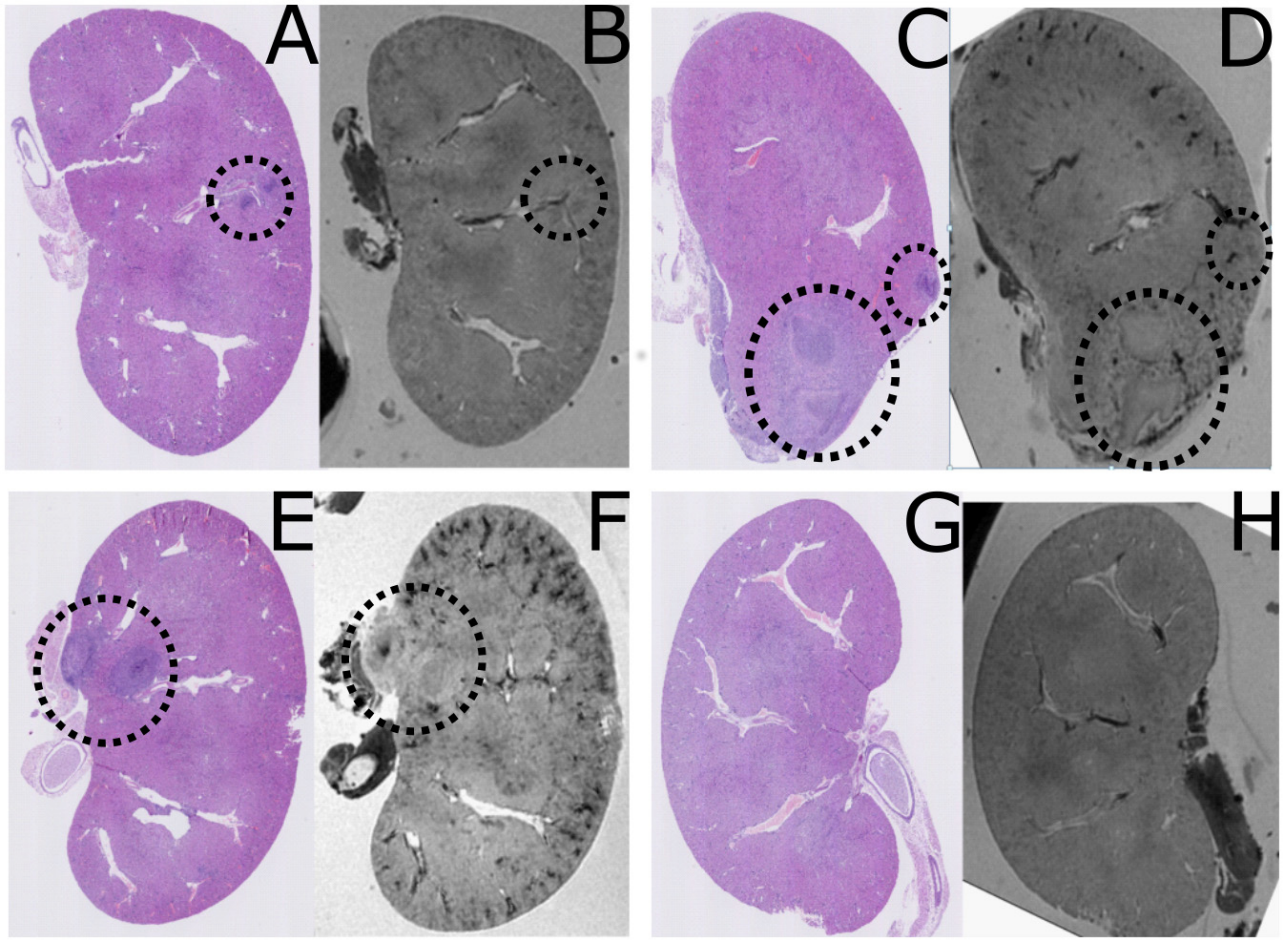
Staphylococcal abscess formation following intravenous infection of 10^7^ CFU *S*. *aureus* in mice. Kidneys were harvested at different time points post infection. Shown are pairs of images of the same kidney section, with the left hand section (A,C,E,G) obtained by histological sectioning of kidneys which has previously undergone MR microscopy (B,D,F,H). A,B show results at 3 days post infection (d.p.i), C,D at 7 d.p.i, E,F at 10 d.p.i. G and H show images obtained from mice injected with PBS as a negative control. Circled regions indicate abscesses. A scale bar is provided.

**Fig 4:**
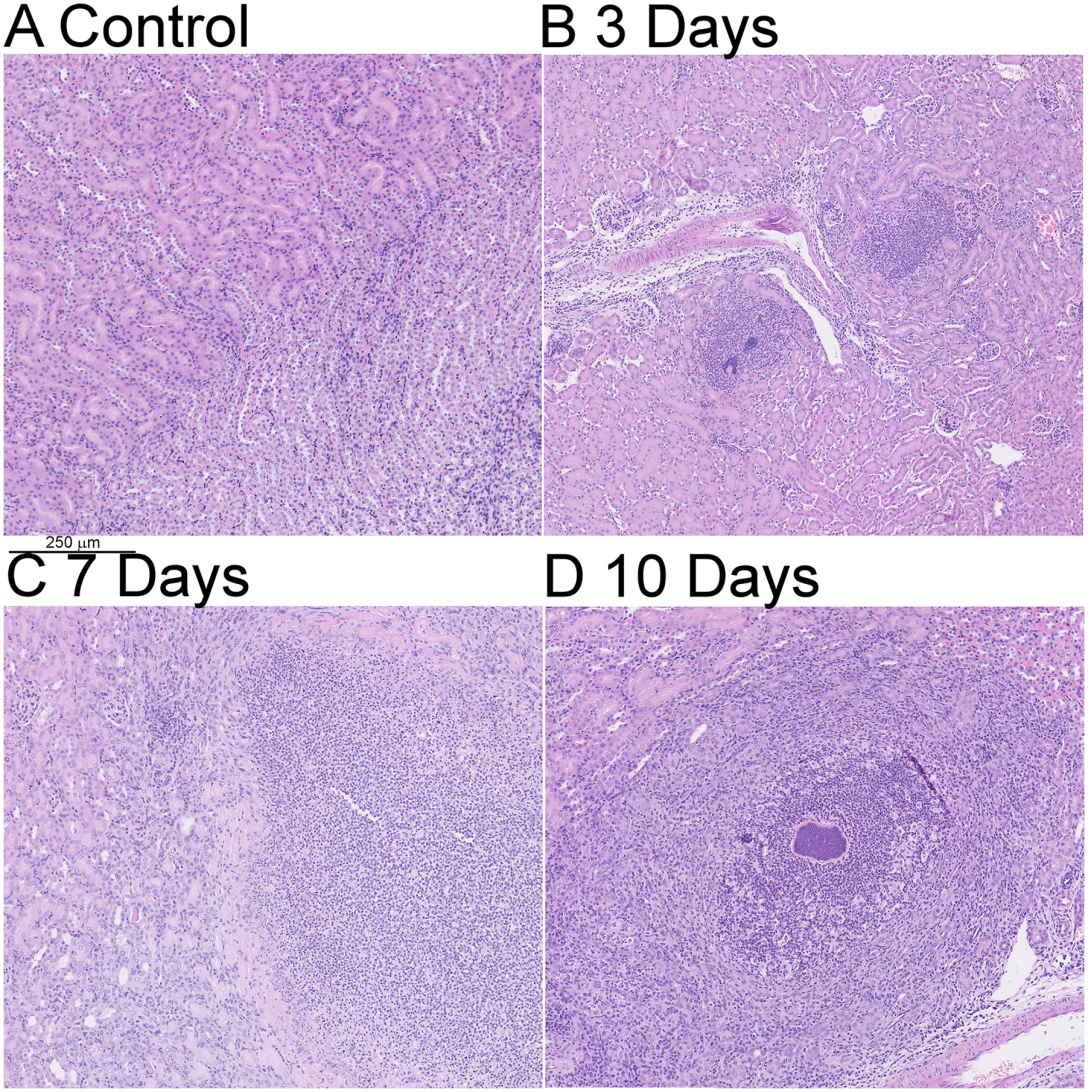
Histological changes distant from macroscopic abscesses. Kidneys were fixed in PFA at various time points after either mock infection (A) or with 10^7^ CFU *S*. *aureus* (B-D). A scale bar is provided.

Since MRI is non-destructive for the organs examined, we were able to recover organs post-scan and perform histological examination to perform MRI:histological correlation on individual organs. In initial inspection, abscesses appeared readily detectable by both techniques, as illustrated in Fig 3. We obtained histology on the test set of 16 kidneys, each with three sections per kidney, and compared these with analysis of MRI images, obtained prior to histologist. Blinded examination by an expert histopathologist (ES) noted 100% concordance between the putative abscesses noted in previous blinded MRI analysis. Importantly, no abscesses were detected by histology which were not also detected by MRI. Histology did, however, identify subtle features specific to infected (as opposed to control) kidneys, including an increase in intra-vessel neutrophils numbers in regions distant to abscesses (Fig 4). We concluded that over the time period 3 to 10 days post infection, MRI appears to be highly sensitive for the detection of *S*. *aureus* abscesses.

#### Comparing multiple slice strategies to analyse volume of abscesses

A key decision in histological analysis is the number of sections to be examined; higher numbers increase sensitivity but also increase cost and labour. We considered the likely performance of MRI based *vs*. histological detection and quantitation of abscesses 3 to 10 days post infection. In view of the high sensitivity of MRI for the detection of abscesses, an ‘electronic histology’ approach was adopted whereby ‘electronic sections’ were cut from a ‘block’ represented by the MRI image. Four slicing strategies, taking 1,3,5 or 10 sections through the long axis of each kidney were considered, specifically: 1 Slice at 50%; 3 Slices: 25, 50, and 75%; 5 Slices: 10, 30, 50, 70, and 90%; 10 Slices: 5, 15, 25, 35, 45, 55, 65, 75, 85, and 95% of the way through the short axis of the kidney. The average area of abscesses detected in ‘electronic sections’ were compared relative to those in the 3D MRI image.

**Fig 5:**
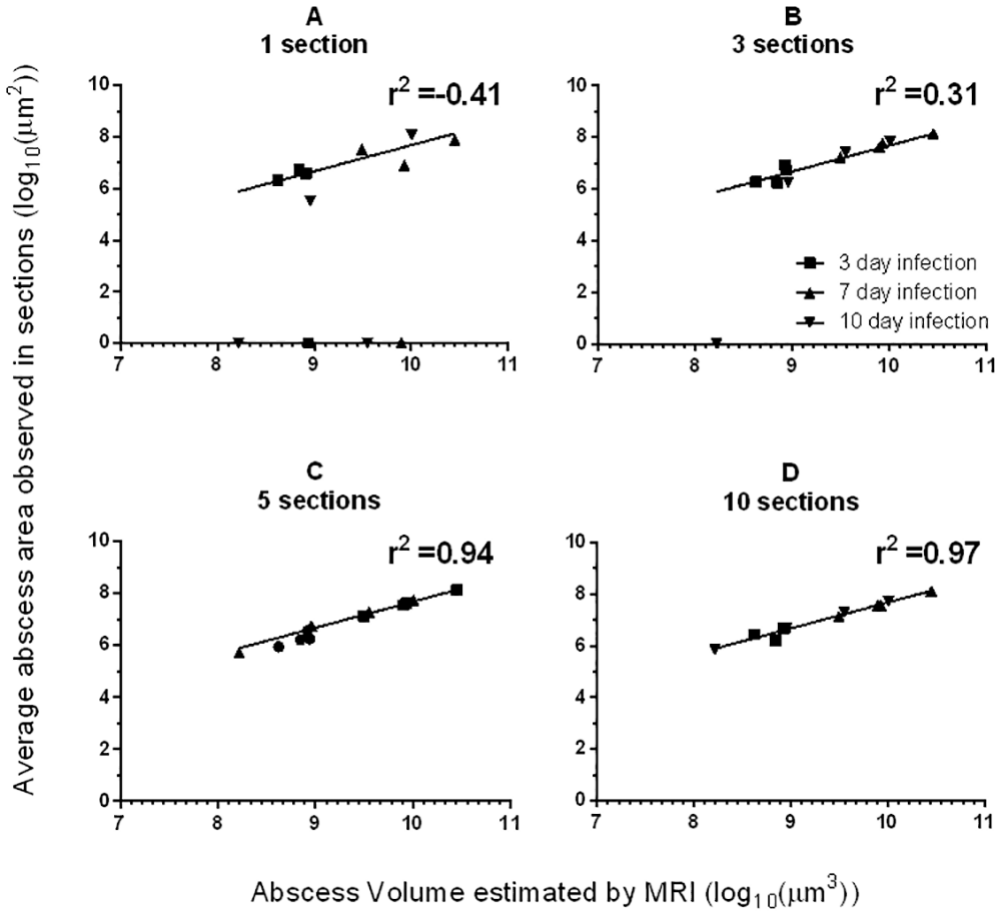
A simulation of various sectioning strategies was performed using the data obtained from MRI scanning. A comparison of the total abscess volume calculated from each kidney and the estimation achieved by an average of abscess areas in sections is depicted for each slicing strategy. (A): 1 slice estimation, (B) 3 slice estimation, (C) 5 slice estimation, (D) 10 slice estimation.

A spherical abscess with radius r has volume proportional to r^3^, while a section through its centre is expected to have area proportional to r^2^. Plotting log (volume) against log (estimated area) is therefore expected to yield a linear relationship, if the section(s) are sufficiently close to the centre of the abscess to approximate the true r. Therefore, as the number of sections rises, the correlation with true abscess volume is expected to improve. A worst case scenario occurs when the abscess is not included in a section at all. Results demonstrated that this expected relationship was observed (Fig 5).

The electronic sectioning technique shows that a single section misses abscesses frequently (4/12 abscesses, 33%), with 1/12 being missed by a three section technique (Fig 5A). As would be expected, correlation between histological and true abscess volume improved as section number rose, with five and ten section strategies offering high correlations with true abscess size (Fig 5C and 5D). We concluded that the MRI technique allowed quantification of abscess volumes.

#### Comparison of bacterial recovery with abscess formation

**Fig 6:**
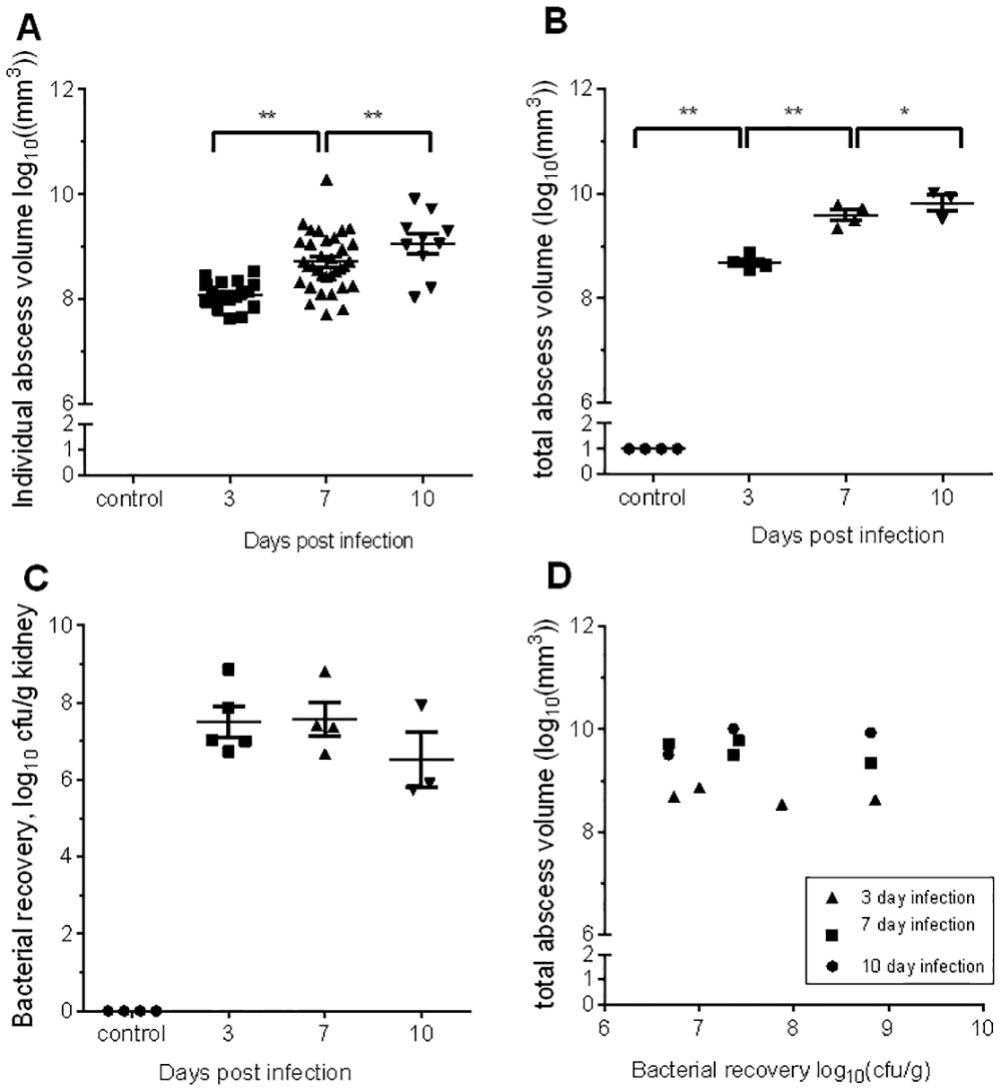
Quantification of abscess growth using MRI. Female Balb/c mice were injected intravenously with 10^7^ CFU *S*. *aureus* Newman. Kidneys were recovered at varying days post infection (DPI). A: The right kidney was fixed in paraformaldehyde, and processed for subsequent MRI analysis. Individual abscess in each kidney (A) and the total volume of abscess present per kidney (B) were calculated. Both the median volume of abscesses detected (A), and the total volume of abscesses (B) increase significantly over time, but (C) bacterial recovery in the left kidney changed little between 3 and 10 days post infection (C,D). Where no abscesses were detected, abscess volume was considered to be 1mm^3^ (Panel B), and when no bacteria were isolated, 1 CFU/g is plotted (Panel C). ** denotes p<0.01, * denotes p<0.05 by Mann-Whitney test.

Analysis of a cohort of mice infected with the same inoculum showed detected abscess number (as assessed by MRI) to increase over time course (Fig 6A). This was also true of abscess volume (Fig 6B). By 10 days, the abscess(es) occupied a substantial part of the renal volume (Fig 6, panel B). This observation is supported by published work which reported abscess diameter estimated from histological sectioning[10]. By contrast, bacterial recovery did not increase over time after day 3 (Fig 6, panel C). Previous experiments conducted using this challenge model show that there is a positive correlation between CFU recovery in the left vs the right kidney of each mouse [26]We concluded that MRI imaging revealed information not discerned from microbiological quantification.

### Evaluation of SrtA vaccine

**Fig 7:**
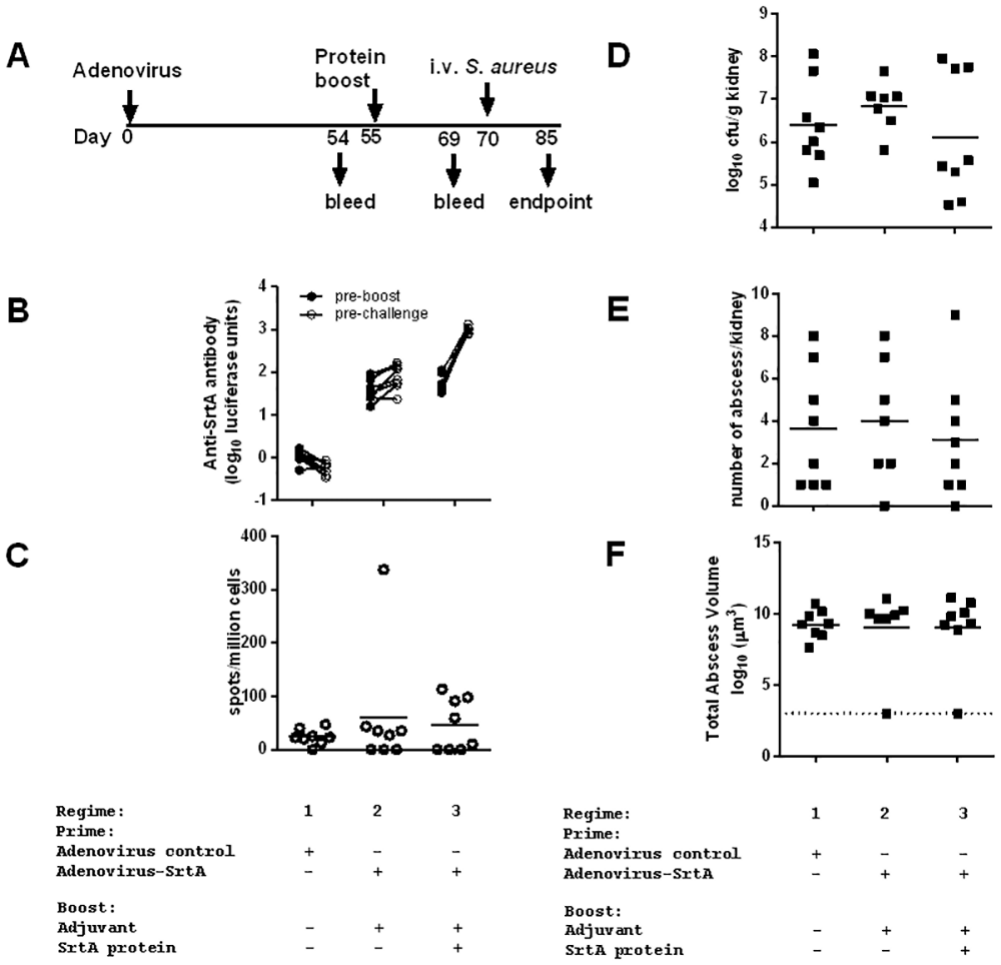
Immunogenicity of Sortase A vaccine regimes. Mice were immunised with either a control regime comprising AdHu5 (Human adenovirus 5) expressing no transgene (Regime 1), or with AdHu5 expressing SrtA (Regime 2), or with the same vector followed 8 weeks later by a SrtA protein boost (Regime 3) (A). (B) Serological assay was performed on serum taken from immunised mice and specific antibody concentration to SrtA is presented in arbitrary luciferase units, calculated as log_10_(Luciferase units- Background) in the LIPS assay used (C) An ELISPOT was performed from blood taken from immunized mice seven days on day 63 post adenoviral priming, or day 7 post protein boost. The difference between groups was not statistically significant. 15 days after infection, (D) bacterial recovery from the left kidney, and renal abscess formation (E,F) assessed by the MR technique were determined.

The use of the technique was illustrated in a study of vaccination against Sortase A (SrtA) [10]. Balb/c mice were vaccinated with adenovirus Hu5 expressing either no exogenous antigen (control) or SrtA, followed by a booster injection with SrtA protein or a vehicle control. This regime (Fig 7A) has previously been used to generate high titres of antibodies against malaria proteins[21]. The vaccine was immunogenic, generating specific antibody responses and weak interferon γ producing T-cells (Fig 7B and 7C). Protective efficacy of the vaccine regime was evaluated by a bacterial challenge. Symptoms scores were assessed daily. One mouse in Group 2 was culled on Day 12 post challenge (day 83 of the experiment) as it had reached the humane endpoint. 7Renal bacterial counts were assessed at day 15. There was no significant difference in bacterial counts between groups (Fig 7, panel D) (ANOVA, p = 0.47), with observed counts similar to those seen at earlier time points in this model (Fig 6C). MRI scanning detected extensive abscess formation in all groups (S4 Video; Fig 7E). Total abscess volume did not differ by vaccine group at this time point (Fig 7F) (ANOVA on log abscess volume, p = 0.54). There was no significant correlation between bacterial load and abscess volume between kidneys (r = 0.11 (95% CI -0.33–0.51)). We concluded the vaccine regime tested was not effective in the prevention of abscess formation following intravenous challenge, and that the MRI technique detected abscess expansion not detected using count-based approaches.

## Discussion

The aim of this study was to establish and validate an MR screening method to quantify abscess size in murine kidneys after challenge with *S*. *aureus*. Using high-resolution MRI (near-isotropic voxel size ~ 25μm), we were able to show that, for abscesses 3 days or later post infection, there is an excellent correlation between histopathological abscess quantification and MRI appearances. Although unable to detect single cells (about 10 cells of about 8 μm diameter would be expected per voxel), independent review by a pathologist indicated that the estimates of abscess formation recovered from MR image analysis was similar to that obtained histologically. There was also a very high level of inter-observer concordance (r = 0.9 for correlation between estimated abscess volumes between two observers). Unlike histological analysis, the entire kidney is imaged; simulation showed that this complete reconstruction allowed more accurate estimation of abscess size than histology, unless 5 or more sections were taken through the kidney.

It has been reported that there is a poor correlation between *S*. *aureus* abscess size and bacterial recovery[10]. The MRI technique confirms this: over the course of ten days, abscesses grew but bacterial recovery in the partner kidney changed little. Thus, abscess size confers additional information relative to bacterial recovery, and highlights the need for a technique that is able to reliably quantify abscess size.

A limitation of the MRI approach is that it does not reveal information on the distribution of cell type(s), bacterial pseudocapsule, or the expression of cell surface or bacterial molecules, although all of which could be monitored by immunohistochemistry. The two approaches are not mutually exclusive, however, histology can be performed following MRI, either on a pre-specified protocol, or targeting areas of interest identified by MRI. While the ex vivo MRI technique precludes investigation of abscess formation serially in the same mouse, it provides superior resolution compared to any *in vivo* MR imaging approach. Use of MRI to detect *S*. *aureus in vivo* has been reported in various models of murine infection [15, 27, 28]. However, future work will explore whether or not this technique can also provide sufficient spatial resolution and contrast *in vivo* to allow for accurate assessment of abscess development in murine renal tissue, allowing sequential monitoring of disease progression [29].

Having established and validated this new technique, its use was illustrated in a study of vaccine efficacy. SrtA, a bacterial enzyme required for abscess formation, was studied[10]. The vaccination regime, which generated antibodies against SrtA, offered no protection in this challenge model: neither the bacterial recovery from the kidneys nor the MRI analysis of the abscess volumes showed any significant difference between the vaccinated and the control group. Explanations might include lack of access of the antibodies to the SrtA enzyme, or lack of function of the antibodies generated.

To conclude, the technique described provides an important tool for enumerating and monitoring abscess growth, and is suitable for use in preclinical vaccination development in mice challenged with intravenous *S*. *aureus*.

## Supporting Information

S1 File Supplementary protocol. This describes the technique used for analysis of the MRI images.
(DOCX)

S1 Fig Layout of samples in S4 Video. This depicts the layout of kidneys in the S4 Video.
(DOCX)

S1 Table Scoring system used for mice challenged intravenously with *S*. *aureus*. This describes the scoring system for mice following intravenous challenge.
(DOCX)

S2 Table Group assignations in S4 Video. (DOCX)

S1 Video MRI video showing a typical set of normal kidneys. The video is of kidneys reconstructed in one plane. The video goes from the top to the bottom of the agar tube containing kidneys which was used for analysis.
(MP4)

S2 Video MRI video showing images of kidneys obtained three days after infection. The video is of kidneys reconstructed in one plane. The video goes from the top to the bottom of the agar tube containing kidneys which was used for analysis.
(MP4)

S3 Video MRI video showing images of kidneys obtained seven- ten days after infection. The video is of kidneys reconstructed in one plane. The video goes from the top to the bottom of the agar tube containing kidneys which was used for analysis.
(MP4)

S4 Video MRI video showing images of kidneys from the sortase A vaccination experiment. The video is of kidneys reconstructed in one plane. The video goes from the top to the bottom of the agar tube containing kidneys which was used for analysis. The groups are as shown in Fig 7, with the addition of three non-infection control kidneys, designated C. For example, in row 8 the kidneys are laid out as in S1 Fig. The group assignations are shown in S2 Table.
(MP4)
