# Supplementary material for: MRI Based Localisation and Quantification of Abscesses following Experimental S. aureus Intravenous Challenge: Application to Vaccine Evaluation
Source: PLoS One. 2016 May 26;11(5):e0154705. doi: 10.1371/journal.pone.0154705 (PMC4881890; doi:10.1371/journal.pone.0154705)
Supplement: S1 File — This describes the technique used for analysis of the MRI images. (DOCX) [file pone.0154705.s001.docx]

Supplementary protocol

1. Open data (usually a stack of .tiff files) with Amira software
2. Set voxel resolution to that of the MR instrument used
3. Apply 3 orthoslices to the data in each of the planes xy, xz and zy
4. Apply a ‘label field’ module to the data
5. Open the segmentation editor
6. Create new materials e.g. ‘abscess #1, abscess #2’ etc.
7. Select areas within the image that appear to be abscess and add these to the desired material
   1. This can be achieved using the paintbrush or lasso tool
      1. For a 3d estimation of the desired material, utilise the paintbrush tool to select sections in each orthoslice which intersect through the centre of the abscess
      2. Apply the selection wrap tool
      3. Add the selected voxels to the material eg abscess #1
8. Once all the desired abscesses have been selected return to the object pool
9. Select the labels module and apply ‘material statistics ‘. This will generate a report of all defined materials within the data set


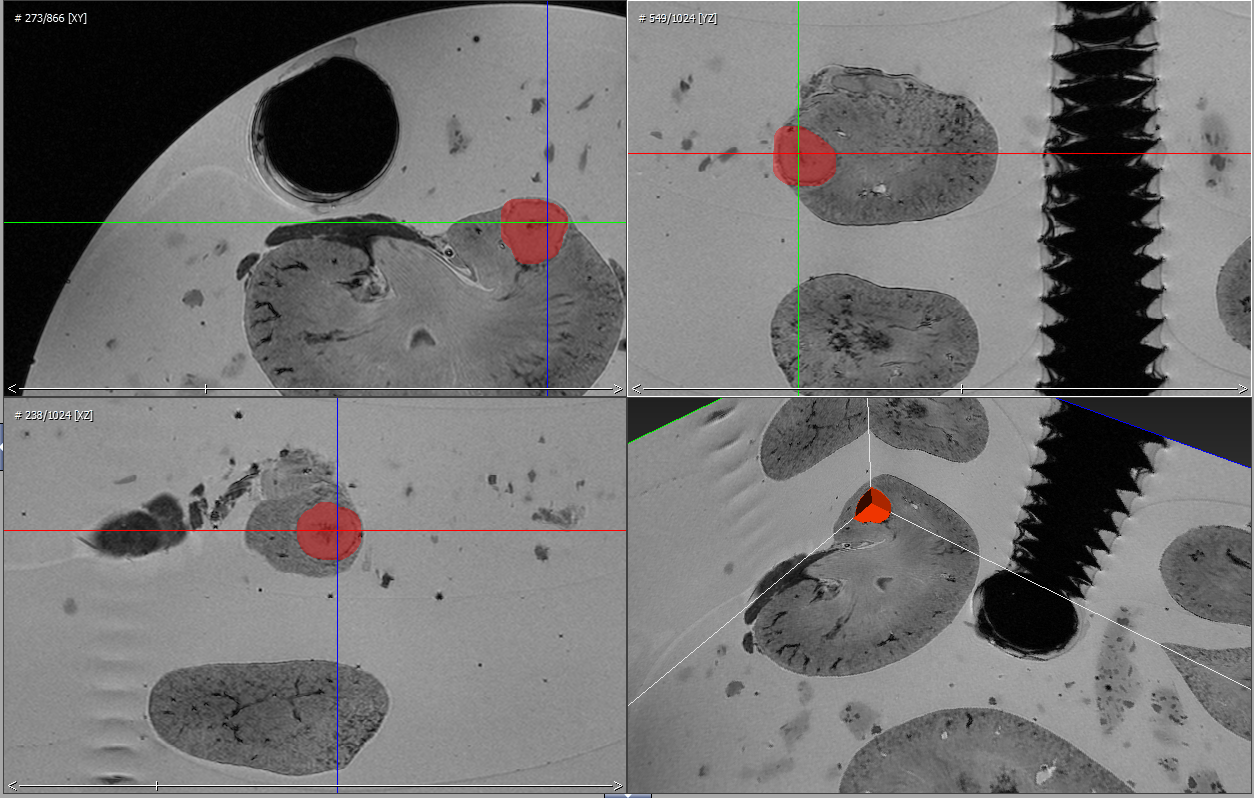


Fig 1. Selection of abscess as a result of step 7. The top left panel shows the xy view, top right shows the yz and bottom left shows the xz view. The bottom right panel shows a 3d simulation of the 3 planes.
