# Supplementary material for: MRI Based Localisation and Quantification of Abscesses following Experimental S. aureus Intravenous Challenge: Application to Vaccine Evaluation
Source: PLoS One. 2016 May 26;11(5):e0154705. doi: 10.1371/journal.pone.0154705 (PMC4881890; doi:10.1371/journal.pone.0154705)
Supplement: S1 Fig — This depicts the layout of kidneys in the S4 Video. (DOCX) [file pone.0154705.s002.docx]

**S1 Figure**

This shows the orientation of the groups in the S4 Video. Please see the S4 Video legend for more details.

C

1

2

3
