# Supplementary material for: MRI Based Localisation and Quantification of Abscesses following Experimental S. aureus Intravenous Challenge: Application to Vaccine Evaluation
Source: PLoS One. 2016 May 26;11(5):e0154705. doi: 10.1371/journal.pone.0154705 (PMC4881890; doi:10.1371/journal.pone.0154705)
Supplement: S1 Table — This describes the scoring system for mice following intravenous challenge. (DOCX) [file pone.0154705.s003.docx]

**Table S1: Scoring system used for mice challenged intravenously with *S. aureus***

|  | Description | Score |
| --- | --- | --- |
| Appearance | Normal | 0 |
|  | General lack of grooming | 1 |
|  | Coat staring, ocular and nasal discharges | 2 |
|  | Piloerection, hunched up | 3 – endpoint |
| Behavior | Normal | 0 |
|  | Slight change; either less or more active than usual | 1 |
|  | Agitated, alert, isolated or significantly less mobile | 2 |
|  | Vocalisation, self-mutilation, unusual aggression or stillness | 3 - endpoint |
| Mouse grimace score* | Orbital tightening | 0-1-2 |
|  | Nose and/or cheek bulge | 0-1-2 |
|  | Ear position | 0-1-2 |
| Arthropathy+ | Normal | 0 |
|  | Decreased movements | 1 |
|  | Decreased movements and either swelling of joints or nodose tail | 2 |
|  | Decrease movements and retracted limb | 3 - endpoint |
| Total score |  | 0-24 |

**According to Langford, D.J., et al., Coding of facial expressions of pain in the laboratory mouse. Nature Methods, 7, 447–449 (2010). +The arthritis scoring system is based on the known manifestations of S. aureus arthritis as described in a series of papers by Tarkowski A and colleagues, e.g Bremell T et al 1995 Infection & Immunity 63: 4185; Bremell T 1992 Infection & Immunity 60: 2976.*
