# Supplementary material for: MRI Based Localisation and Quantification of Abscesses following Experimental S. aureus Intravenous Challenge: Application to Vaccine Evaluation
Source: PLoS One. 2016 May 26;11(5):e0154705. doi: 10.1371/journal.pone.0154705 (PMC4881890; doi:10.1371/journal.pone.0154705)
Supplement: S2 Table — (DOCX) [file pone.0154705.s004.docx]

| Row | 9am | 12 noon | 3pm | 6pm | Position in tube |
| --- | --- | --- | --- | --- | --- |
| 8 | C | I | 2 | 3 | Bottom (start of video) |
| 7 | C | I | 2 | 3 |  |
| 6 | C | I | 2 | 3 |  |
| 5 | 3 | 3 | 2 | 1 |  |
| 4 | 1 | 2 | 3 | 3 |  |
| 3 | 2 | 2 | 1 | 3 |  |
| 2 | 3 | 1 | 2 | 2 |  |
| 1 | Empty | Empty | Empty | Empty | Top (end of video) |
